# Mortality in patients with cirrhosis admitted to the ICU: time to rethink strategies?

**DOI:** 10.1186/cc14465

**Published:** 2015-03-16

**Authors:** A Vaz, M Eusebio, A Antunes, A Sousa, P Perez, R Ornelas, C Granja, H Guerreiro

**Affiliations:** 1 Centro Hospitalar do Algarve, Faro, Portugal

## Introduction

Cirrhotic patients admitted to the ICU are usually regarded as having a particularly poor prognosis when compared with other groups of critically ill patients. The aim of our study was to evaluate the prevalence, case mix and outcomes of patients with cirrhosis admitted to the general ICU of a nontransplant center.

## Methods

Data were collected from a running ICU database. We studied cirrhotic patients admitted to the ICU between January 2013 and November 2014.

## Results

A total of 30 patients with cirrhosis were admitted, accounting for 3% of total ICU admissions. Mean age was 54.5 years, with a male preponderance (76.7%). The main cause for cirrhosis was alcohol (53.3%), followed by alcohol plus chronic hepatitis C virus (HCV) infection (20%) and HCV virus infection alone (13.3%). The most common causes for admission were sepsis/septic shock (26.7%), surgical (23.4%), gastrointestinal bleeding and hepatic encephalopathy (16.7% each). At admission, these patients presented an average Model for End-Stage Liver Disease score of 23.5 ± 10.4 with 70% classified as grade C in the Child-Pugh score; mean Acute Physiology and Chronic Health Evaluation (APACHE II) 29.2 ± 8.7 and new Simplified Acute Physiology Score (SAPS II) 62.7 ± 29. Regarding organ failure at admission, the mean Sequential Organ Failure Assessment score was 12.8 ± 4.5. The ICU mortality of these patients was 43.3% and hospital mortality was 53.3%. The variables at admission that related significantly with ICU mortality were: all scores described except for Child-Pugh score, bilirubin, the International Normalized Ratio, creatinine, bicarbonate, lactate, pH and the use of renal replacement therapy during the ICU stay (*P *< 0.05). The mortality rate of cirrhotic patients was superior to the general ICU mortality (43% vs. 26%). However, patients with cirrhosis presented significantly higher severity scoring systems (APACHE II; SAPS II) at admission compared with noncirrhotics, with high prevalence of organ dysfunction as assessed by SOFA score.

## Conclusion

The high severity of disease in conjunction with the high mortality rate observed in this group of patients should make us consider the possible benefits of earlier referring/admission to the ICU, ideally before multiorgan failure arises. On the other hand, in nontransplant centers where cirrhotic patients constitute a small percentage of total ICU admissions, the complexity and peculiarities of the management of these patients should prompt their early transfer to a specialized center.

